# Danshenol A Mediates the Proliferation and Differentiation of Adipocytes in Thyroid-Associated Ophthalmopathy

**DOI:** 10.2174/0118715303382831250712200617

**Published:** 2025-07-24

**Authors:** Yuting Chen, Jie Min, Xiali Yu, Haixiang Ni, Yamei Jin

**Affiliations:** 1 Department of Endocrinology, The First Affiliated Hospital of Zhejiang Chinese Medical University (Zhejiang Provincial Hospital of Chinese Medicine), Hangzhou, China;; 2 Department of Endocrinology, LongHua Hospital, Shanghai University of Traditional Chinese Medicine, Shanghai, China;; 3 Department of Rheumatology, the First Affiliated Hospital of Zhejiang Chinese Medical University (Zhejiang Provincial Hospital of Chinese Medicine), Hangzhou, China

**Keywords:** Thyroid-associated ophthalmopathy, danshenol A, IGF-1R, PI3K/AKT signaling, intraorbital adipose tissues, orbital preadipocytes apoptosis

## Abstract

**Introduction:**

An increase in the intraorbital adipose tissue is the main pathological feature of thyroid-associated ophthalmopathy (TAO). IGF-1R activates PI3K/AKT signaling and accelerates adipogenesis. Pingmu decoction has been demonstrated to promote orbital adipocyte apoptosis; however, less is reported regarding the action mechanism of Danshenol A (DA), a single active ingredient of *Salvia miltiorrhiza* (Danshen). Accordingly, this study aimed to investigate the role and association of DA and IGF-1R in the proliferation and lipid accumulation of orbital adipocytes.

**Methods:**

Primary human orbital preadipocytes were chosen and authenticated using immunofluorescence. Cells were treated with the IGF-1R agonist ginsenoside Rg5, IGF-1R overexpression plasmid, dexamethasone (Dex), and/or DA, after which cell proliferation and differentiation were assessed by cell counting kit-8 (CCK-8), oil red O staining, real-time quantitative polymerase chain reaction, and Western blot assays.

**Results:**

Orbital preadipocytes showed positive expression of Pref-1. Treatment with IGF-1R agonist, as well as Dex, promoted orbital adipocyte viability and lipid accumulation, and increased the expression of adiponectin and leptin. It was observed that the overexpression of IGF-1R boosted PI3K/AKT activation and elevated PPARγ and C/EBPα expressions. Importantly, DA reversed the effects of IGF-1R on cell viability, lipid accumulation, and the PI3K/AKT signaling pathway.

**Discussion:**

This study, for the first time, revealed the molecular mechanism by which DA regulates orbital fat metabolism through targeted inhibition of the IGF-1R/PI3K/AKT signaling axis. Notably, IGF-1R overexpression partially counteracted the inhibitory effect of DA, suggesting that this component has multi-target regulatory characteristics.

**Conclusion:**

This study not only reveals a new mechanism by which DA treats TAO but also provides theoretical support for the treatment of adipose metabolism.

## 1 INTRODUCTION

Thyroid-associated ophthalmopathy (TAO) is an autoimmune disease commonly associated with Graves’ disease [1], characterized by primary clinical manifestations that include inflammation of the soft tissues of the eye, eyelid recession, and eyeball protrusion, which can significantly impact patients appearance and visual function [2]. Genetic predisposition and environmental factors are contributors to the development of TAO [3]. The current treatment of TAO mainly relies on glucocorticoid therapy, immunosuppressive therapy, and monoclonal antibody drugs; however, their limited efficacy and numerous side effects may contribute to an unfavorable patient prognosis [4]. Pathologically, TAO primarily features increased intraorbital adipose tissue, hypertrophic fibrosis of the extraocular muscles, and inflammatory lesions [5, 6]. Further studies on the proliferation and differentiation of orbital adipocytes may be contributive to the treatment of TAO.

Various active components of Traditional Chinese Medicine (TCM) have shown potential therapeutic benefits in *in vitro* and animal studies of TAO [7, 8]. Pingmu decoction has demonstrated therapeutic efficacy in improving TAO symptoms with fewer adverse effects clinically [9, 10]. Specifically, it can promote orbital adipocyte apoptosis [9] and also inhibit the differentiation of preadipocytes into mature adipocytes in patients with TAO [10]. However, Pingmu decoction is a compound formula, and its specific component that can regulate adipose differentiation needs to be further explored. Here, we selected and analyzed Danshenol A (DA), an active ingredient of *Salvia miltiorrhiza* in Pingmu decoction, which has been shown to exhibit strong inhibitory activity against rat eye lens aldose reductase [11]. The related effects of DA have been less discussed in the literature. Notably, DA attenuates oxidative stress and apoptosis by regulating mitochondrial dysfunction and repairing mitochondrial structure/function [12]. Therefore, we mainly focused on the function and mechanism of DA when treating TAO with Pingmu decoction.

In addition, the insulin-like growth factor-1 receptor (IGF-1R) is a widely studied autoantibody associated with TAO [13, 14], and the IGF-1R blocking antibody is used as a therapeutic agent for TAO [15]. IGF-1 can accelerate adipogenesis by specifically binding to IGF-1R and activating the PI3K signaling pathway [16]. According to the Herb database, PPARG, one of the downstream effectors of PI3K signaling, is a possible target for DA. Other scholars have suggested that PPARG is an adipogenesis-related marker [17]. Furthermore, a previous study reported that the overexpression of IGF-1R is accompanied by dysregulation of the IGF-1R axis, which is connected to other pathways regulating inflammation, such as fibrosis and the extracellular matrix, and thus plays a role in patients with thyroid eye disease [18]. In this study, we used human orbital preadipocytes to probe into the role of DA in cell proliferation and adipogenic differentiation, and its relationship with the IGF-1R/PI3K axis.

## 2 MATERIALS AND METHODS

### 2.1 Cells and Characterization

Primary human orbital preadipocytes were gifted by the Central Laboratory of Longhua Hospital, Shanghai University of Traditional Chinese Medicine.

Orbital preadipocytes (3 × 10^7^/L) were seeded in 6-well plates and fixed for 10 minutes at room temperature using 4% paraformaldehyde (AR-0211, Dingguo, China). Later, cells were blocked with 2% bovine serum albumin (4240GR005, BioFroxx, China) and incubated with Pref-1 antibody (dilution 1:200; ab119930, Abcam, UK) at 4 °C overnight and FITC secondary antibody (dilution 1:500; ab150113, Abcam, UK) for 30 minutes. DAPI (ab228549, Abcam, UK) was used to stain nuclei for 2 minutes, followed by examination under a fluorescent inverted microscope (DMi8, Leica, Germany).

### 2.2 Treatment and Transfection

Orbital preadipocytes were treated with ginsenoside Rg5 (Rg5, HY-N0908, MCE, USA), an IGF-1R agonist dissolved in DMSO, at a concentration of 20 μM for 36 hours, with untreated cells serving as the negative control (NC). In the second part, IGF-1R overexpression plasmid (C05003) was synthesized by GenePharma (China), with an empty vector as NC. In the dexamethasone (Dex) group, to induce the maturation and differentiation of preadipocytes in orbital preadipocytes, cells were treated with 0.5 mM 3-isobutyl-1-methylxanthine (I106812, Aladdin, China), 1 μM Dex (D137736, Aladdin, China), and 10 μg/ml insulin (I657407, Aladdin, China) for 48 hours. Following IGF-1R overexpression plasmid transfection using the Lipofectamine 3000 kit (L3000001, Invitrogen, USA), cells were treated with DA (HY-122917, MCE, USA).

### 2.3 Cell Counting Kit-8 (CCK-8) Assay

Cell viability was assessed using the Cell Counting Kit-8 (CCK-8) (CA1210, Solarbio, China). Briefly, different groups of orbital preadipocytes (5 × 10^3 cells/well) were seeded in a 96-well plate and then mixed with CCK-8 solution (10 μL) for 3 hours. After that, optical density at 450 nm was recorded by a SpectraMax ABS plus microplate reader (Molecular Devices, China) for viability analysis.

### 2.4 Oil Red O Staining

Oil red O staining kit (C0157) was purchased from Beyotime (China). After the cells were fixed using 4% paraformaldehyde, an appropriate amount of staining wash solution was added to cover the cells for 20 seconds. The cells were stained with oil red O staining solution for 15 minutes, and the nuclei were then re-stained using hematoxylin staining solution (C0107, Beyotime, China). A Ti2 microscope (Nikon, Japan) was used to record lipid droplet formation.

### 2.5 Real-Time Quantitative PCR (RT-qPCR)

TRIzol reagent (15596-026, Invitrogen, USA) and a NanoDrop ND-LITE spectrophotometer (Thermo Scientific, USA) were used for RNA harvesting and concentration determination, respectively. RNA reverse transcription was achieved by RevertAid First Strand cDNA Synthesis Kit (K1622, Thermo Scientific, USA). SYBR Green qPCR Master Mix (HY-K0501A, MCE, USA) equipped with a real-time PCR system (ABI7500, Applied Biosystems, USA) was employed for PCR amplification. The primers were as follows (5’-3’). Adiponectin: TGCTGGGAGCTGTTCTACTG, TACTCCGGTTTCACCGATGTC; Leptin: TGCCTTCCAGAAACGTGATCC, CTCTGTGGAGTAGCCTGAAGC; IGF-1R: TCGACATCCGCAACGACTATC, CCAGGGCGTAGTTGTAGAAGAG; GAPDH: GGAGCGAGATCCCTCCAAAAT, GGCTGTTGTCATACTTCTCATGG.

### 2.6 Western Blot

Cells were lysed using RIPA Lysis Buffer (20-188, Millipore, USA) for protein collection, and then the protein concentration was determined using a BCA kit (BCA1, Sigma-Aldrich, USA). Proteins were separated using 10-12% SDS-PAGE gels and transferred to polyvinylidene difluoride membranes (FFP28, Beyotime, China). The membranes were incubated with appropriate primary antibodies overnight at 4 °C, and goat anti-rabbit or goat anti-mouse secondary antibodies (ab205718/ab205719) for 2 hours at room temperature. ECL luminescence reagents (GS1551, Bjbalb, China) were exploited to recognize blot signals. The primary antibodies (Abcam, UK) used are as follows: IGF-1R (1/1000, ab39675, 156kDa); C/EBPα (1/1000, ab40761, 43kDa); PPARγ (1/1000, ab178860, 58kDa); Akt (1/500, ab8805, 60kDa); phosphor (p)-Akt (1/1000, ab38449, 56kDa); PI3K (1/1000, ab302958, 127kDa); p-PI3K (0.5 µg/ml, ab278545, 84kDa); GAPDH (1/1000, ab8245, 37kDa).

### 2.7 Statistical Analysis

Statistical analysis was performed using GraphPad Prism 8.0 (GraphPad Prism, USA). Measurement data were expressed as mean ± standard deviation. Comparisons between two groups of data were performed by an independent samples *t*-test, and comparisons among multiple groups were made by one-way ANOVA with Tukey’s test. Data with *P*<0.05 were regarded as statistically significant.

## 3 RESULTS

### 3.1 IGF-1R agonist Rg5 Promoted Orbital Preadipocyte Viability and Lipid Accumulation

**Fig. (1):**
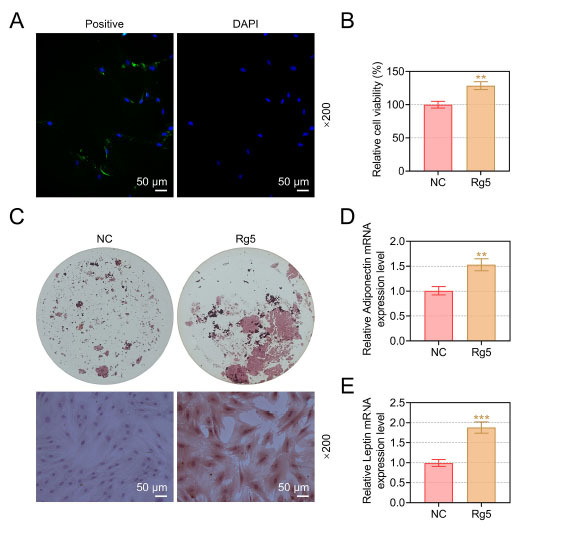
IGF-1R agonist Rg5 promoted proliferation and lipid accumulation in orbital preadipocytes. (**A**) Identification of preadipocytes by immunofluorescence, scale bar: 50 μm, magnification: ×200. (**B**) Cell viability detection by CCK-8 assay. (**C**) Determination of cellular lipidogenic differentiation by Oil red O staining, scale bar: 50 μm, magnification: ×200. (**D-E**) Quantification of adipogenesis-related markers, adiponectin and leptin, by RT-qPCR. CCK-8: cell counting kit-8; RT-qPCR: real-time quantitative polymerase chain reaction. **P<0.01, ***P<0.001 *vs*. NC (negative control), n=3.

Primary human orbital preadipocytes were authenticated by immunofluorescence, which showed Pref-1 positivity (Fig. **1A**). Next, we treated the cells with Rg5 as an IGF-1R agonist. Compared with NC group, Rg5 group displayed promoted orbital preadipocyte viability (*P* < 0.01, Fig. **1B**). Oil red O staining data demonstrated that Rg5 increased lipid droplet formation in the cells (Fig. **1C**). Mechanistically, the expressions of adiponectin and leptin were higher in the Rg5 group than in the NC group (*P* < 0.01, Figs. **1D**-**E**).

### 3.2 DA reversed the IGF-1R-Mediated Regulation of the Viability and Differentiation in Human Orbital Preadipocytes

**Fig. (2):**
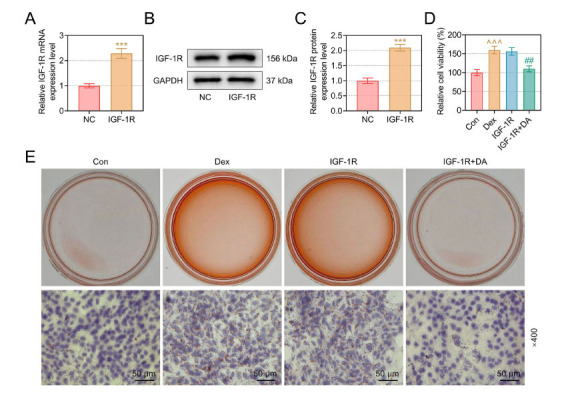
Danshenol A (DA) reversed the effect of IGF-1R overexpression on viability and differentiation in human orbital preadipocytes. (**A-C**) The transfection rates of IGF-1R overexpression plasmid (RT-qPCR (**A**) and Western blot (**B-C**)). (**D**) The viability of orbital adipocytes in Con (Control), Dex (dexamethasone group), IGF-1R, and IGF-1R+DA groups (CCK-8). (**E**) Adipose differentiation in each group (oil red O staining), scale bar: 50 μm, magnification: ×400. ***P<0.001 *vs*. NC (negative control vector); ^^^^^*P*<0.001 *vs*. Con; ^##^*P*<0.01 *vs*. IGF-1R, n=3.

DA, an active ingredient of Danshen extracted from the drug monomer in Pingmu decoction, was selected as our research target. IGF-1R overexpression significantly upregulated IGF-1R mRNA and protein levels (*P* < 0.001, Figs. **2A**-**C**). Functional experiment results showed that in Dex or IGF-1R group, orbital adipocyte viability was enhanced; however, DA reversed the effect of IGF-1R overexpression on orbital adipocyte viability (*P* < 0.01, Fig. **2D**). Similarly, Dex or IGF-1R overexpression both increased lipid droplet formation in human orbital preadipocytes, but DA offset the effect of IGF-1R overexpression on cellular lipogenic differentiation (Fig. **2E**).

### 3.3 DA improved Viability and Differentiation of Human Orbital Preadipocyte Through the IGF-1R/ PI3K/AKT Signaling Pathway

**Fig. (3):**
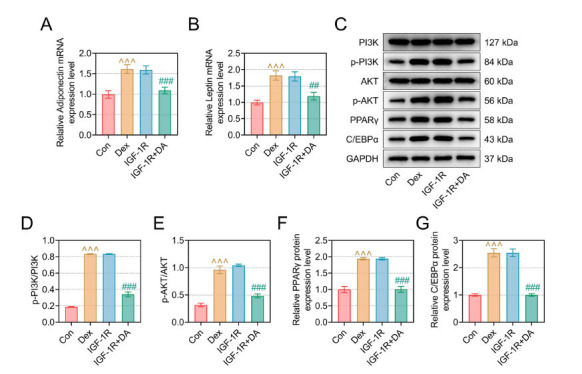
DA offset the effects of IGF-1R overexpression on adipose differentiation-related factors and PI3K/AKT signaling pathway-related proteins. (**A-B**) Effects of DA and IGF-1R overexpression on the expressions of adiponectin and leptin (RT-qPCR). (**C-G**) Levels of PI3K, p-PI3K, AKT, p-AKT, PPARγ, and C/EBPα proteins (Western blot). ^^^^^*P*<0.001 *vs*. Con; ^##^*P*<0.01, ^###^*P*<0.001 *vs*. IGF-1R, n=3.

To further clarify the mechanism of DA-IGF-1R in human orbital preadipocytes, we quantified the expression of adipogenic markers. Dex or IGF-1R overexpression increased the expression of adiponectin and leptin. DA suppressed the upregulation of the two markers and attenuated the role of IGF-1R overexpression (*P* < 0.01, Figs. **3A**-**B**). In addition, p-PI3K/PI3K, p-AKT/AKT, PPARγ, and C/EBPα expressions were elevated by Dex or IGF-1R overexpression, and DA dampened the IGF-1R overexpression-induced activation of PI3K/AKT signaling pathway (*P* < 0.001, Figs. **3C**-**G**).

## 4 DISCUSSION

TAO is one of the most common autoimmune clinical diseases, causing ocular protrusion [19, 20]. Investigating the proliferation and differentiation of TAO preorbital adipocytes may lead to the discovery of more suitable therapeutic agents or targets. Here, we proved that DA has an inhibitory effect on the viability and lipogenic differentiation of orbital preadipocytes by reducing IGF-1R/PI3K/AKT expression. Our study further elucidated part of the pathomechanism of Pingmu decoction in TAO treatment and suggested that DA might be a useful single therapeutic agent for delaying TAO progression.

Pre-adipocyte fibroblasts in orbital tissues can differentiate into adipocytes under suitable conditions and play a crucial role in adipose tissue cell proliferation [21]. We used human orbital preadipocytes as subjects, whose cell membranes express Pref-1. Reportedly, orbital preadipocytes have also been employed in the study of apoptosis induction *in vitro* using Pingmu decoction [10]. It has been documented that IGF-1R is a glycoprotein heterotetramer belonging to the tyrosine kinase receptor family, which has important roles in pro-mitosis, pro-transformation, and anti-apoptosis, and mediates the inflammatory response in TAO orbits [22, 23]. Rg5, which has a high affinity to IGF-1R, has been reported as an IGF-1R agonist in angiogenesis studies [24]. Rg5 has a high safety profile and a variety of biological functions, including anti-inflammatory, antidiabetic, and neuro- and cardio-protective properties [25]. Loss of IGF-1R is associated with a reduction in TAO-related adipogenesis [26]. We unveiled the promoting effects of an IGF-1R agonist and the impact of its overexpression on the viability and lipogenic differentiation of orbital preadipocytes, which was in agreement with the previous studies.

Great attention has been paid to the role of traditional Chinese medicine in treating TAO [7, 27, 28]. Hai *et al*. reviewed the therapeutic effects of thujaplicin, curcumin, and gypenosides, and linked Chinese medicine to the inhibition of orbital adipose fibroblast proliferation and inflammation [7]. Berberine blocks nuclear factor-κB signaling to control orbital fibroblast adipogenesis, inflammation, hyaluronan production, and fibrosis [29]. DA, one of the constituents of *Salvia miltiorrhiza*, has been confirmed to alleviate mitochondrial dysfunction and reduce ROS production [12]. In atherosclerosis studies, DA suppresses TNF-α-induced monocyte adhesion [30]. Additionally, DA can inhibit the release of inflammatory factors [31]. The PI3K/AKT pathway and the MAPK pathway are considered to be the primary signaling mechanisms of Yifei Tongluo granules against idiopathic pulmonary fibrosis, and the granules contain DA active ingredients [32]. Herein, we found a possible ameliorative effect of DA on TAO, as evidenced by enhanced viability and differentiation of orbital preadipocytes, as well as downregulation of adiponectin and leptin.

Unlike other ocular diseases, TAO activates the IGF-1R and PPARγ signaling pathways [33]. IGF-1R is enriched in the early stages of lipogenesis, and PI3K/AKT signaling is activated in TAO [34]. The inhibitory effect of disulfiram on lipid accumulation is also accompanied by a decrease in PPARγ, C/EBPα, and other proteins [35]. It has been reported that glucocorticoids, including Dex, may reduce hyaluronic acid in orbital cells [36]. Here, we used Dex in combination with insulin or other drugs to induce maturation and differentiation of orbital preadipocytes. Additionally, both Dex, in combination with insulin or other drugs, and IGF-1R can induce differentiation, and their differentiation-inducing abilities are comparable. It was found that IGF-1R/PI3K/AKT expression in mature adipocytes was enhanced by IGF-1R treatment. Importantly, DA reversed the effect of IGF-1R. This indicates that DA might regulate the IGF-1R/PI3K/AKT pathway to improve viability and differentiation of human orbital preadipocytes. Interestingly, IGF-1 activates the PI3K/AKT pathway by binding to IGF-1R [37]. Pretreatment with linsitinib can reduce the phosphorylation of IGF-1R, PI3K/AKT, and extracellular signal-regulated kinase (ERK) in orbital fibroblasts of TAO patients induced by IGF-1 [38]. A previous study demonstrated that, upon activation by its ligand (IGF-1), IGF-1R is localized to the nucleus of orbital fibroblasts through the ADAM17 signaling pathway [39]. Furthermore, IGF-1 is a major inflammatory factor and a critical index for diagnosing TAO [13]. In the complex pathophysiological process of TAO, extensive interactions may occur among various signaling pathways. Therefore, more research is needed to fully reveal these interrelationships. The binding affinity of DA to IGF-1R will be determined using Surface Plasmon Resonance (SPR) technology to optimize the dosing strategy. An in-depth exploration of the role of DA, IGF-1R, and PI3K/AKT signaling pathways in TAO is of great significance for the diagnosis and treatment of the disease.

However, this study has some limitations. First, in this study, only *in vitro* cell tests were conducted, and for the verification of the PI3K/AKT signaling pathway, no activator or inhibitor was used. Second, the results lack further verification through *in vivo* experiments. Furthermore, the effect of DA on physiological levels of IGF-1R in a wild-type background has not been measured, which requires further study.

## CONCLUSION

Overall, it was found that DA, an active ingredient of Pingmu decoction, inhibits IGF-1R-induced viability and lipogenic differentiation of orbital preadipocytes by regulating the expression of the PI3K/AKT pathway. Our study may provide insight into the mechanism of DA for treating TAO and suggest that DA is a potential IGF-1R blocker for inhibiting the malignant progression of orbital preadipocytes.

## Data Availability

The data and supportive information are available within the article.

## LIST OF ABBREVIATIONS

TAO: Thyroid-Associated Ophthalmopathy
DA: Danshenol A
Dex: Dexamethasone
CCK-8: Cell Counting Kit-8
TCM: Traditional Chinese Medicine
NC: Negative Control
IGF-1R: Growth Factor-1 Receptor
